# The distribution of four trace elements (Fe, Mn, Cu, Zn) in forage and the relation to scrapie in Iceland

**DOI:** 10.1186/1751-0147-52-34

**Published:** 2010-05-21

**Authors:** Tryggvi Eiríksson, Hólmgeir Björnsson, Kristín Björg Gudmundsdóttir, Jakob Kristinsson, Torkell Jóhannesson

**Affiliations:** 1 Agricultural University of Iceland, Department of Animal and Land Resources, Keldnaholt, 112 Reykjavík, Iceland; 2 Actavis Group, Clinical Research Department, Reykjavíkurvegur 80, 220 Hafnarfjördur, Iceland; 3 Department of Pharmacology and Toxicology, University of Iceland, Hofsvallagata 53, 107 Reykjavík, Iceland

## Abstract

**Background:**

Previous studies indicated that the iron (Fe)/manganese (Mn) ratio in forage of sheep was significantly higher on scrapie-afflicted farms than on farms in other scrapie categories. This study was conducted to examine whether Fe and Mn in forage of sheep varied in general according to the scrapie status of different areas in the country. Copper (Cu) and zinc (Zn) were also included because of a possible relation to scrapie.

**Methods:**

The country was subdivided into seven Areas (I-VII). Three Areas (I, IV, VII) were designated scrapie-free (never diagnosed or eradicated) and three as scrapie-endemic (II, III, VI); status of Area V was taken as unsettled. Of the harvest 2007 1552 samples were analysed from 344 farms all over the country, mostly grass silage from plastic bales (>90%) and from the first cut (70% or more). Results were expressed as mg kg^-1 ^dry matter.

**Results:**

Fe varied enormously from less than 100 mg kg^-1 ^to 5000 mg kg^-1^. Mn varied nearly thirtyfold (17-470 mg kg^-1^). Fe concentration was significantly lower in Area I than in Areas II, V and VI. Mn concentration was significantly higher in Areas I, IV and VII than in Areas II, III, V and VI. The Fe/Mn ratio was significantly less in Area I than in the other areas (except Area IV). Mean Cu concentration was 6.6-8.3 mg kg^-1 ^and the mean Zn concentration was 24-29 mg kg^-1^. They differed significantly in some areas.

**Conclusions:**

1) Fe tended to be in lower amounts in sheep forage in scrapie-free than in endemic areas; 2) Mn was in higher amounts in forage in scrapie-free than endemic areas; 3) the Fe/Mn ratio was lower in scrapie-free than in endemic areas; 4) the Fe/Mn ratio may possibly be used as an indicator of scrapie status; 5) Cu and Zn in sheep forage were not related to scrapie; 6) further study on the role of Fe and Mn in the occurrence of scrapie in Iceland is needed.

## Background

*Jóhannesson et al*. [1] have previously found significantly higher concentration of manganese (Mn) in the forage from scrapie-free farms in scrapie free counties (Category 1) than on scrapie-free farms (Category 2), scrapie-prone farms (Category 3) or on scrapie-afflicted farms (Category 4) in scrapie-affected counties in Iceland. Mn was also in significantly higher concentration in samples from farms in Category 2 than in Category 4 but not in samples from farms in Category 3. Although the Mn concentrations were found to vary highly in the samples they were in general in the same range as is considered as normal for plants [2]. The idea was subsequently promulgated that high levels of Mn in the forage of sheep, albeit in the normal range, might have a protective effect against the occurrence of clinical scrapie and the effect could possibly be confined to the cellular border of the gastrointestinal tract [1,3]. Later *Gudmundsdóttir et al*. [4] demonstrated the existence of a certain reciprocality between the iron (Fe) and Mn concentrations in the forage of sheep. These authors found the Fe/Mn ratio significantly higher in forage samples from farms in Category 4 than in the other categories. Thus the results would indicate that high amounts of Fe in the forage might somehow premise the occurrence of clinical scrapie.

Scrapie has during recent years been diagnosed sporadically on casual farms in especially two areas, one in the north and another in the southern part of the country, while most other areas have been essentially free of scrapie for about 20 years at least (cf. *Gudmundsdóttir et al*. [5]). Thus the main aim of the present study was to investigate whether there is any possible connection between the Fe/Mn ratio in the forage of sheep in general and the occurrence of clinical scrapie in these areas. For this purpose the country was subdivided in seven areas according to their appreciated scrapie status. In these seven areas about 1550 samples of forage of the 2007 harvest were collected on more than 300 farms and subjected to Fe and Mn trace metal analysis.

The study also included determination of copper (Cu) and zinc (Zn) in the samples. This was due to the experimental findings that Cu might facilitate the endocytosis of the prion protein, and that Zn might be in higher concentration in the forage from farms in Category 1 as compared to farms in the other categories [3].

## Materials and methods

### Subdivision of the country in seven areas

**Figure 1:**
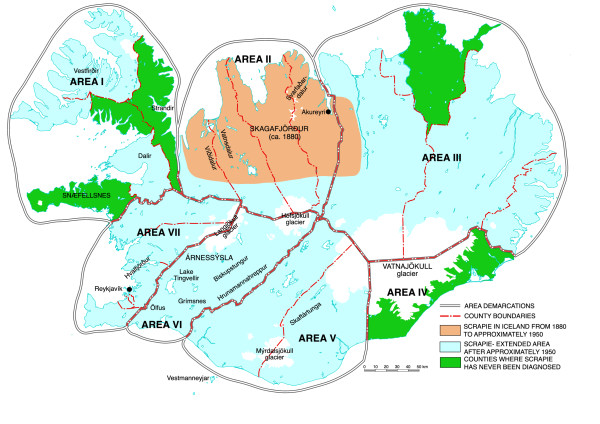
**Scrapie in Iceland. Subdivision of the country in seven areas according to their appreciated scrapie status and the inclusive counties indicated**. Scrapie was, from its presumed origin in Skagafjörður around 1880, confined to a part of northern Iceland until ca. 1950 (orange). It has since spread patchily to greater or lesser parts of all counties (blue) except for four and a major naturally demarcated part of the fifth (green). Three of these counties are found in Area I and the fourth is Area IV. The farms where scrapie has been diagnosed from the year 2000 (incl.) are located in Area II (16 farms), Area VI (11 farms) and in Area III (2 farms). Scrapie has not been diagnosed in Areas I and VII for 20-25 years and not after about 1990 in Area V. The scrapie-free counties and the large green area in the north-east corner of the country are the main areas in the country used to provide healthy lambs to restock formerly scrapie-afflicted farms.

The country was subdivided in seven areas according to their appreciated scrapie status (Figure 1).

*Area I *encompasses the north-western peninsula (The Vestfirðir) including the region Strandir with numerous sheep farms. To the south the area includes the Dalir and the Snæfellsnes Counties. Three of the four scrapie-free counties (scrapie never diagnosed) are found in this area. In this area scrapie was first diagnosed in a locality on the south-western part of the Vestfirðir Peninsula in 1953 and it was most likely brought there due to illegitimate transport of sheep from afflicted areas. Although the disease in these regions was considered to be of an unusually grave nature (several animals per flock presenting clinical symptoms) and spread patchicly to a considerable degree it has seemingly been eradicated in this area (not found in the Vestfirðir Peninsula after 1985 and in the Dalir not after 1988).

*Area II *includes four counties in the north where scrapie was diagnosed before approximately 1950 and where it has been found regularly up to the present date (diagnosed on 16 farms from the beginning of the year 2000) in different regions like the Víðidalur, Vatnsdalur, Skagafjörður and Svarfaðardalur. On two of these farms scrapie was diagnosed in January 2009. It is presumed that scrapie in Iceland originated in the Skagafjörður region around 1880 with the import of sheep of foreign stock.

*Area III *is a large area in eastern Iceland with a variable scrapie record. Thus a large chunk of a county on the north-eastern corner has remained scrapie-free (the scrapie-free region is demarcated to the west by a torrential glacial river; only four samples were received from this region) whereas scrapie has repeatedly been diagnosed from about 1968 or before on farms in both the north-western and the southernmore regions of this area (scrapie diagnosed on 2 farms from the year 2000 inclusive).

*Area IV *represents a county in south-eastern Iceland, south of the large glacier Vatnajökull, where scrapie has never been diagnosed.

*Area V *represents two counties in southern Iceland. In the westernmost county scrapie has only been diagnosed a few times and not after 1984. In the eastern part, especially in the region Skaftártunga, scrapie has been diagnosed a few times from 1984 but as far is known not with certainty after 1990 or thereabout. On the whole the data pertinent to this area are bound with some uncertainty. Included in this area are three farms in Category 3 (see below and Table 1).

**Table 1:** Numbers of farms and forage samples (in parentheses) in each scrapie category (Cat.) in the seven areas and the total numbers (see also the text and Figure 1).

Number of farms and samples				
**Area**	**Cat. 1**	**Cat. 2**	**Cat. 3**	**Total**

I	8 (32)	16 (67)	0	24 (99)
II	0	100 (416)	21 (83)	121 (499)
III	0	46 (188)	10 (31)	56 (219)
IV	5 (12)	0	0	5 (12)
V	0	54 (257)	3 (13)	57 (272)
VI	0	57 (306)	4 (12)	61 (318)
VII	0	20 (133)	0	20 (133)

Total numbers:	13 (46)	293 (1367)	38 (139)	344 (1552)

*Area VI *is the county Árnessýsla where scrapie has often been diagnosed from about 1975 and up to the present date (diagnosed on 11 farms from the year 2000 inclusive). The most scrapie afflicted regions in this area have been the Biskupstungur and Hrunamannahreppur but the disease has also been diagnosed in the Grímsnes and Ölfus regions.

*Area VII *is the south-western part of the country. Almost all of the samples collected in this area came from farms in two counties located to the north of the Hvalfjörður as sheep farming, except for some amateur sheep keeping, is only sparsely found south of this fjord. Scrapie was first diagnosed in the northern part of this area in 1951 in sheep that had been transported from an afflicted area (Area II). Scrapie has not been diagnosed in Area VII after 1983. Thus scrapie has seemingly been eradicated in this area.

From the foregoing it thus seems that Areas I, IV and VII can be considered as scrapie-free, Areas II, III and VI as scrapie-endemic whereas the status of Area V seems somewhat uncertain. Most of the data on the occurrence and dispersion of scrapie in Iceland are from the review article of *Sigurdarson *[6] or obtained from him by personal communication (Jan. 2009).

It should be noted that of the 29 farms where scrapie was diagnosed from the beginning of the year 2000 to the end of January 2009 an atypical form of the disease (Nor98) was found on one farm in Area II and on two farms in Area VI. The diagnosis of scrapie in the central nervous system of sheep in Iceland is based on the work of *Thorgeirsdóttir *and her colleagues [7-9], and is centered at the Keldur Institute for Experimental Pathology, Reykjavík.

### Samples and farms

As a part of an annual farming routine agricultural advisers, the respective farmers or others, collected samples of forage from farms in Iceland of the 2007 harvest for the determination of macroelements (Na, K, Ca, Mg, P and S), protein and energy. The samples were sent to the Department of Animal and Land Resources at the Agricultural University of Iceland. There the authors got access to the samples for determination of the trace elements Fe, Mn, Cu and Zn. These trace elements were subsequently analysed in 1552 samples (after exclusion of several samples due to visible contamination or other defects) from a total of 344 farms located in the seven areas (cf. Figure 1; Table 1). Of the samples 1427 were grass silage taken from ordinary silage bales wrapped in plastic, 15 from extra large silage bales, 63 from old-type ensilage and 53 from dry hay (dry matter content > 80%). Sixty-eight per cent of the samples were of the first cut (mowing), about 13% of the second cut but for the remaining samples (about 19%) it was not stated explicitly whether they were of the first or second cut. The relative number of samples in the last category was highest in Area I (60%) while the relative number of samples of the second cut was lowest in this area (6%). Although the sampling was not done by the authors or on their behalf and the sample collection was not as homogenous as that previously compiled by the authors with the procedure described by *Jóhannesson et al*. [1] the samples in the present study were generally of good quality.

There were three categories of farms: *Category 1*: Farms located in counties where scrapie has never been diagnosed. *Category 2*: Farms never afflicted by scrapie, or afflicted and restocked prior to 1960, but located amongst scrapie-prone or scrapie-afflicted farms in scrapie-affected counties. *Category 3*: Farms afflicted by scrapie after 1980 and afterwards restocked with healthy sheep. Farms in Category 3 are referred to as *scrapie-prone*. It should be noted that no samples were received from *scrapie-afflicted *farms (Category 4; scrapie recently diagnosed).

Most of the farms were either sheep farms or mixed sheep and cattle farms but a few farms were cattle farms or horse farms only. The number of forage samples per farm was on average about 4.5. From a few farms only one sample was received. Fourteen farms sent more than ten samples each. The highest number of samples was from the country sites of the Agricultural University at the large farm complex Hvanneyri and vicinity in Area VII. From Area IV only twelve samples (from five farms and of the first cut) were received.

Numbers of samples and farms in the seven areas and the three categories are shown in Table 1. More than one third of the farms were located in Area II and almost one third of the samples were received from these farms. For a comparison less than 10% of the farms were located in Area I with also less than 10% of the samples coming from this area.

### Grass species

According to *Thorvaldsson *[10] the six most common grass species on Icelandic grass fields are: *Poa pratensis *(Kentucky bluegrass), *Festuca rubra *(red fescue), *Phleum pratense *(Timothy), *Deschampsia caespitosa *(tufted hairgrass), *Agrostis sp*. (bentgrass) and *Poa annua *(annual bluegrass). The author divided the country into four regions: east, south, west and north and found differences between regions as four of the species are concerned, with the extreme figures most often in the region south. These data may also apply to our material but we did not analyse the samples with regard to botanical composition.

*Thorvaldsson *[10] emphasized that the proportion of dioctyledon weeds is low in Icelandic grass fields. It is also of importance that clover has been extremely rare in seed mixtures in Iceland for many years.

### Preparation of samples and metal analyses

When the samples were received at the laboratory they were mixed and homogenized. Next a part of the homogenized samples was dried at 60°C in a forced air oven for approximately 24 hours. After being stabilized at room temperature for one or two days the analytical samples were milled through a 1 mm screen in a hammer mill. Around 0.18 mg of a sample was accurately weighed into a special glass test tube. Samples were then digested by boiling in 5 ml of concentrated HNO_3 _(Merck Suprapur; Merck KgaA, Darmstadt, Germany) overnight and subjected to analysis.

About 60 samples were digested at each time. Blank samples and two reference samples were included in every run to confirm accuracy of analysis. The reference samples used were our own reference grass sample and certified reference material (Leaves of Poplar NCS CC 73350, China National Analysis Center for Steel, China, supplied by LGC Protochem, Borås, Sweden); three of each in every set of samples. Analyses were then carried out by ICP optical emission spectrometry using a Spectroflame D sequential instrument (Spectro, Analytical Instruments GmbH, Kieve, Germany). The results of individual forage samples were, respectively, the means of three ICP analytical measurements. All values are corrected for dry matter and are expressed as mg kg^-1 ^dry matter.

The determinations were performed at the Department of Animal and Land Resources, The Agricultural University of Iceland, at Keldnaholt, Reykjavík.

### Statistical analyses

The trace metal contents of forage samples were analysed statistically in a mixed effects model with *Areas *as a fixed effects factor, using the Reml (Residual Maximum Likelihood) analytical model in Genstat [11]. *Farms *are the units for classification into scrapie categories and are, therefore, the basic random units and repeated samples on a farm are subsampling. Regional variations or trends within *Areas *are in the model represented by districts, *Hreppur*, as a random effects factor. The random variation is thus at three levels, *Hreppur, Farms *within *Hreppur *(*Hreppur/Farms*), and *Samples *within *Farms *(*Farms/Samples*). The distribution of Fe and Mn was skew and the results were transformed logarithmically in order to approach the normal distribution. Estimated mean values were transformed back on the original scale and they are suitably interpreted as estimates of medians although this is strictly true only when the log-distribution is symmetric.

The SNK (Student-Newman-Keuls') multiple range test [12] was used in a modified form to evaluate the statistical significance of differences among Areas at the *5% level *of significance. The standard errors are based on a combination of variance components rather than a least squares estimate of variance so that they are not associated with a known number of degrees of freedom (df). As the number of *Hreppur*, the highest order of classification, is rather high (118) the test is not sensitive to the degrees of freedom and the number 120 was used as an approximation. First he means are ordered from the largest to the smallest (or *vice versa*). The procedure then proceeds stepwise, beginning with the difference between the largest and the smallest over the standard error of difference. Critical values for the number of means in the range between and including the means being tested are obtained from the tabulated upper percentage points of the studentized range with 120 degrees of freedom for error. When a difference is declared significant, the lowest (highest) mean is excluded and means now at the end of the range compared. In the SNK test a new critical value is sought for the number of means in the range actually being tested. If the difference is declared nonsignificant the comparisons with the highest are stopped and the means between and including the means last compared constitute a range of nonsignificant differences. If there were means outside this range the procedure is repeated with comparisons with the second highest and so on until the lowest (highest) value is included in a nonsignificant range or it is declared significantly different from the nearest mean. In the present data replication, and consequently standard errors, was unequal. The procedure was modified so comparisons may be continued within a nonsignificant range if there are smaller standard errors of difference within the range.

The difference between any two scrapie categories was tested by adding them to the model as a fixed effect and restricting the analyses to those *Areas *where both categories were found together.

## Results

**Figure 2:**
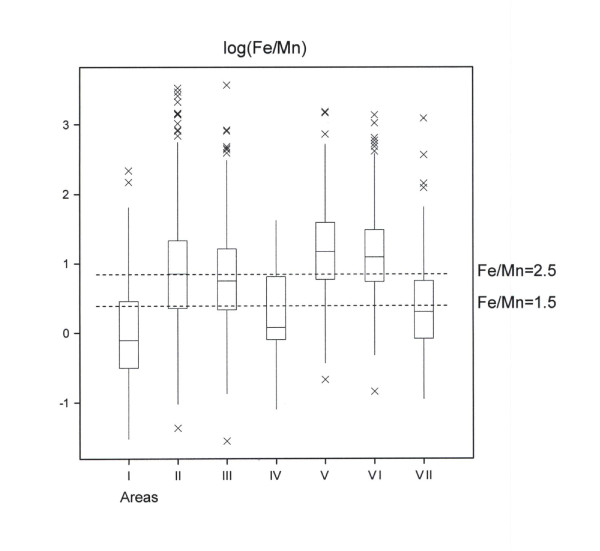
**Boxplot of log(Fe/Mn) in 7 different areas in Iceland (see Figure 1 and text)**. The boxes span the middle 50% of the data and the horizontal line within each box indicates the median. Whiskers extend to the minimum and maximum values up to a distance of 1.5 times the interquartile range and more outlying points are shown as distinct points. The dotted horizontal lines indicate the interval of favorable ratio of Fe/Mn from 1.5 to 2.5 in plants.

The results are presented in Figure 2, Tables 2 and 3 and in the text. The few samples from Area IV were not included in the statistics of significance in Table 2. The inclusion of district variation (*hreppur*) within Areas as a component of the random variation was of particular importance for proper interpretation of the Mn results. Further elaboration of the variation of results will be published elsewhere.

**Table 2:** The means of Fe, Mn, Cu and Zn analyses in forage from farms in the seven areas with the means of the calculated Fe/Mn ratios included.

Area	log (Fe)	log (Mn)	log (Fe/Mn)	Cu	Zn
I	4.91 b	4.79 a	0.10	6.66 bc	24.1 bc
II	5.23 a	4.43 b	0.78 b	7.42 ab	24.8 c
III	5.15 ab	4.28 bc	0.86 ab	6.90 bc	24.8 c
IV	5.14 -	4.76 -	0.38 -	8.27 -	28.7 -
V	5.38 a	4.19 c	1.20 a	6.58 c	28.0 ab
VI	5.28 a	4.19 c	1.10 ab	7.52 ab	28.9 a
VII	5.24 ab	4.63 a	0.65 b	8.14 a	26.0 abc
SED means	0.118	0.106	0.159	0.376	1.13
(excl. Area IV)					

Log (Fe): the logarithm of the mean iron concentrations; Log (Mn): the logarithm of the mean manganese concentrations;

Log (Fe/Mn): the logarithm of the mean iron/manganese ratios; Cu: mean concentrations of copper (mg kg^-1^); Zn: mean concentrations of zinc (mg kg^-1^). SED: Standard error of difference. Means marked with the same letter, a, b or c, constitute groups of non-significant differences at the α = 0.05 (5%) level of significance. Area IV is not included in the construction of non-significant ranges and the SED is the mean of values excluding comparisons with Area IV.

**Table 3:** The means of Fe, Mn, Cu and Zn analyses in forage from farms in Categories 2 and 3 with the means of the calculated Fe/Mn ratios included (see also legend to Figure 2).*

Category	log(Fe)	log(Mn)	log(Fe/Mn)	Cu	Zn
2	5.27	4.27	1.00	7.09	26.6
3	5.19	4.32	0.87	7.24	26.3
SED	0.086	0.068	0.095	0.27	0.99

*The total number of samples was 1308 and the farms were located in Areas II, III, V and VI.

SED: Standard error of difference.

### Iron

On the whole the Fe concentration was found to vary greatly. Thus the Fe concentration was below 100 mg kg^-1 ^(40-100 mg kg^-1^) in 204 samples (13%) and it was in the highest range (1000-5000 mg kg^-1^) in 37 samples (2.4%) (10 of these samples were in excess of 2000 mg kg^-1^). Transformed on the scale of measurement the results in Table 2 indicated that the median Fe concentration was 136 mg kg^-1 ^in Area I and it was in the range 171-217 mg kg^-1 ^in the other areas. The mean Fe concentration was significantly lower in Area I than in Areas II, V and VI. Other differences of statistical significance were not observed.

### Manganese

The Mn concentration varied nearly thirtyfold, lowest 16.4 mg kg^-1 ^and highest 467 mg kg^-1^. Mn was below 40 mg kg^-1 ^in 140 samples (9%). Mn concentration was above 200 mg kg^-1 ^in 62 samples (4%). The estimated median Mn concentration was highest in Areas I, IV and VII (range 103-120 mg kg^-1^). In the other areas (II, III, V, VI) the estimate was below 100 mg kg^-1 ^(66-84 mg kg^-1^). The mean Mn concentration was significantly higher in Areas I and VII than in Areas II, III, V and VI, and the difference between Area II on the one hand and Areas V and VI on the other was also significant.

### The Fe/Mn ratio

The adjusted means for log(Fe/Mn) are approximately the same as the difference log(Fe) - log(Mn). The differences that occur are due to the reduced weight of repeated samples in the analytical model (Table 2). The adjusted mean for Area I, 0.10 (Table 2), corresponds to the median value Fe/Mn = 1.1 and it was found to be significantly less than in all the other areas (except Area IV). In Areas II, IV and VII the estimated median was in the range 1.5-2.2 (with the highest value in Area II). Areas II and VII were found to differ significantly from Area V with the highest value (3.3). In Areas III and VI the medians were in the range 2.4-3.0. The distribution of results is shown in Figure 2.

There are some apparent differences between the medians in Figure 2 and the adjusted means in Table 2. In particular the median value in Area VII is low compared to the results in Table 2. This discrepancy is due to the fact that in this area the distribution of samples on farms was particularly uneven (the many samples from the Agricultural University farms are shown individually on Figure 2 but they have low weight each in the results of Table 2).

### Copper

The Cu concentration varied about fifteenfold, lowest 1.9 mg kg^-1 ^and highest 29 mg kg^-1^. Sixty-one samples had Cu concentration lower than 4.0 mg kg^-1 ^(4%). The Cu concentration was in the range 10-30 mg kg^-1 ^in 120 samples (7.7%). The Cu results were approximate to the normal distribution. The lowest mean concentration of Cu (Table 2) was in Areas I, III and V (range 6.6-6.9 mg kg^-1^) and the highest in Areas IV and VII (about 8.2 mg kg^-1^). The mean concentration was significantly higher in Area VII than in Areas I, III and V. It was also significantly higher in Areas II and VI than in Area V.

### Zinc

The lowest Zn concentration was 3.2 mg kg^-1 ^and the highest 79 mg kg^-1^. Twelve samples (0.8%) had lower concentration than 10 mg kg^-1 ^and 13 samples (0.8%) higher than 50 mg kg^-1^. The Zn results were approximate to the normal distribution and the mean values are shown in Table 2. The mean Zn concentration was in the range 24-29 mg kg^-1 ^in all areas with the lowest concentration (< 25 mg kg^-1^) in Areas I, II and III and the highest (28 mg kg^-1^) in Areas IV, V and VI. The difference between these two groups of Areas was found significant except for Area IV and the difference between Areas I and V.

Models including dry matter digestability (DMD), a property that is closely related to the maturity of forage, and the classification of samples into cuts were evaluated. All four elements were in higher concentration in the second cut as compared with the first cut and the Fe/Mn ratio accordingly remained the same. DMD decreases with the maturity of the forage which has variable effect on the four elements (Cu and Zn decreased, Mn increased, Fe was the same and consequently the Fe/Mn ratio decreased with maturity). The effect of adjustment for these two variables on the standard error of differences was, however, most often very small and had insignificant effects on the Area means.

### Comparison of farms in Categories 2 and 3

As is shown in Table 1 no farms included in Category 3 were found in Areas I, IV and VII. Farms in Categories 2 and 3 were found in Areas II, III, V and VI. Results from farms in these two categories were compared. The logarithmic means of Fe and Mn determinations, the logarithmic means of the Fe/Mn ratios and the mean Cu and Zn concentrations did not differ significantly in sheep forage between these categories of farms (Table 3).

## Discussion

The term "scrapie-prone" has a special reference to the fact that in recent years many cases of scrapie have been observed sporadically on casual farms in three areas (Areas II, III and VI) where scrapie had been diagnosed previously, the flocks culled and the farms subsequently restocked with healthy sheep in accordance with governmental rules. Before 1960 scrapie was occasionally misdiagnosed as the lentiviral infection visna (eradicated in the sixties). Furthermore the information on the occurrence of clinical scrapie is in general often fragmentary before that time. It should also be noted that systematic, preventive measures against scrapie (including culling of flocks, quarantine periods etc.) were first legally enforced just prior to 1980. Thus these two years have been used as cut-out times in this study. The designation of three areas in the county as scrapie-free (Areas I, IV, VII) and other three areas as endemic with scrapie (Areas II, III, VI) has also been outlined above (see Materials and methods).

*Hardarson et al*. [13] found the Fe concentration especially low in forage (first cut 2003) from the Vestfirðir Peninsula (120-140 mg kg^-1^) and from the Snæfellsnes County and the scrapie-free county representing Area IV (70-103 mg kg^-1^). Although the results of these authors on Fe from some other localities (e.g. the Dalir) are apparently at variance with the data presented here their results are nevertheless in support of the notion that the Fe concentration is in general the lowest where the likelihood for occurrence of scrapie is either the least or it has never existed (Figure 1; Table 2). In this context it should be noted that Fe concentrations around 1000 mg kg^-1 ^indicate that either the plants were suffering from Fe poisoning [14] or the forage samples were somehow contaminated from unknown extraneous sources. Concurrent determination of aluminium might have revealed whether the samples were contaminated with Fe of earthy origin or not.

In our study the median Mn concentration was above 100 mg kg^-1 ^in Areas I, IV and VII and it was below 100 mg kg^-1 ^in the other areas. In the study of *Hardarson et al*. [13] the Mn concentration was also high in the Vestfirðir Peninsula, the Snæfellsnes County, the Dalir County and Areas IV and VII (on average 140-185 mg kg^-1^) whereas the Mn concentration was most often lower in other regions. Together these results indicate that the lower levels of Fe in the forage in scrapie-free areas (Areas I, IV and VII) are reciprocated in higher levels of Mn resulting in lower Fe/Mn ratios than in the endemic areas (II, III, VI) (Figure 2; Table 2). *Gudmundsdóttir et al*. [4] have as previously mentioned found the same reciprocality between Fe and Mn in sheep forage resulting in a significantly higher Fe/Mn ratio on scrapie-afflicted farms (Category 4) than on farms in the other categories. The possibility may thus exist that the Fe/Mn ratio in sheep forage may be of some value, at least, as an index of the likelihood for the occurrence of clinical scrapie.

The Fe and Mn concentrations and the Fe/Mn ratios were not found to differ significantly between farms in Categories 2 and 3 (Table 3). In the case of Mn these results are in accordance with the previous results of *Jóhannesson et al*. [1] who found that the Mn concentration was not significantly higher in forage on farms in Category 2 than on farms in Category 3. As the Fe concentrations and the Fe/Mn ratios are concerned the present results are furthermore in concert with the earlier work of *Gudmundsdóttir et al*. [4]. Unfortunately the present sample collection, as is already mentioned, did not include any forage samples from farms in Category 4 (scrapie recently diagnosed).

Mn and Cu concentrations were statistically the same in the blood of ewes on scrapie-free, scrapie-prone and scrapie-afflicted farms (scrapie recently diagnosed) [15]. Although only one or a few animals usually have clinical symptoms in a stricken flock when culled, most often 20-40% of the asymptomatic sheep show pathological changes characteristic of scrapie in the central nervous system [[8]; Chief Veterinary Officer, personal communication]. It was therefore concluded that the possible protective effect of high concentration of Mn in the forage against the occurrence of clinical scrapie might rather be confined to the gastrointestinal tract, which is considered the main port of entry for the prion protein in the sheep, than to any other internal organs. These authors emphasized, however, that variables like seasonal changes and pregnancy might significantly affect the concentration of Mn and Cu in the blood of sheep [15].

The normal prion protein (PrP^c^) is secreted from the endoplasmic reticulum through the Golgi apparatus to the plasma membrane where it is tethered to the surface by a glycosylphosphatidylinositol anchor (GPI-anchor). The pathological prion protein (PrP^sc^) is also assumed to be anchored to the membrane in the same way [16,17]. The formation of the GPI-anchor in the endoplasmic reticulum may involve glycosyl transferases that have a special or unique requirement for Mn as a cofactor [18,19]. High Mn concentration in the forage could hypothetically increase the attachment of the prion protein (PrP^c ^and PrP^sc^) to cell membranes in the gastrointestinal tract and thus retard, or prevent, their entry through the mucosal epithelium. This idea gains in essence support from previous work showing that glycosylation of the prion protein has a kind of protective effect on its conversion to the pathological protein (PrP^sc^) [20,21]. The concentration of Mn in forage was, as far is known, almost always in the socalled normal range for plants [2]. The postulated preventive effect of Mn against scrapie is thus obviously rather biochemical than toxic in nature.

In plants the ratio between Fe and Mn (Fe/Mn ratio) should be in the range 1.5 to 2.5. As the antagonism between Mn and Fe is a well documented interaction in higher plants ratios lower than approximately 1.5 would mean dominance of Mn over Fe whereas the reverse is the case if the ratio approximates or exceeds 2.5 (Figure 2). At extreme high and low ratios the plants might suffer from Fe and Mn toxicity, respectively [2]. Apart from the antagonism of Fe and Mn in plants these metals most likely display antagonistic as well as synergistic effects in animals [22,23]. As far as the prion protein is concerned new evidence indicates that PrP^c ^is an Fe-binding protein and redox Fe may have a fundamental role in the conversion of PrP^c ^to PrP^sc ^[24].

*Basu et al*. [24] have shown in *in vitro *experiments with human neuroblastoma cells expressing PrP^c ^that redox Fe (like FeCl_2_) may induce the conversion of the normal prion protein to a PrP^sc^-like form. Furthermore, depletion of Fe from prion disease-affected human and mouse brains reduced the amount of PrP^sc ^fourfold to tenfold indicating that generation, propagation and stability of PrP^sc ^are modulated by the redox levels of Fe. The results also indicated that glycosylated PrP^c ^is in the presence of redox Fe less accessible to conversion to PrP^sc ^than free or unglycosylated PrP^c^. If similar conditions should reign in the gastrointestinal tract of sheep after normal ingestion of forage it could explain why high Fe content is related to the occurrence of clinical scrapie.

The work of *Hesketh et al*. [25] deserves mentioning. These authors performed several experiments with scrapie in sheep and bovine spongiform encephalopathy in cattle. In experimental scrapie they found that the concentration of Mn (and to some extent of Cu also) increased in the blood whether the animals developed scrapie or not. Thus sheep with the genotype ARR/ARR, considered resistant to classical scrapie, also showed higher levels of Mn in the blood in the course of the experiment. The authors therefore concluded that the elevation of Mn does not result from specific pathological changes but is a consequence of the infection. In this context it should be mentioned, as is already referred to above, that stressful situations like pregnancy may significantly affect the levels of Mn and Cu in the blood of sheep [15].

*Pauly & Harris *[26] and *Sigurdsson et al*. [27] have shown that Cu may facilitate the endocytosis of the prion protein and administration of Cu chelator might significantly delay the onset of prion disease in experiments with mice. Thus it could be expected that high amounts of Cu in sheep forage might be related to the occurrence of scrapie. This was not borne out unequivocally by our study in so far as the mean Cu concentration was lowest in Areas I, III and V but highest in Areas IV and VII. On the whole the Cu concentration was, however, somewhat lower and more variable than in a previous study based on forage samples from farms in Categories 1-4 [1]. Thus about one in every twenty samples had a Cu concentration lower than around 5 mg kg^-1 ^which is the approximate critical concentration in plants [2]. In the study of *Hardarson et al*. [13], as well as in this study, the mean Cu concentration in forage was found to differ significantly between various parts of the country.

In a previous study the concentration of Zn was significantly higher in the forage from scrapie-free farms in scrapie-free counties than in the forage from farms in other categories [3]. This could not be substantiated in the present study as the mean Zn concentration in Area I was in the lowest range along with results for Areas II and III. The Zn concentration was also significantly higher in Area VI than in the three above mentioned areas (Results; Table 2). Thus it is not logical to assume that high amounts of Zn in forage are related to low incidence of scrapie. However, the results of the present study show lower levels of Zn than previously [3,13] and indicate, in accordance with the earlier studies, that the Zn concentration in forage of sheep in Iceland might be lower than optimal. In the survey from 2003 [13] the differences between areas were on the whole less than in the present study although statistically significant differences were found. The ranking of areas was also different 2003. Thus such studies should preferably be based on more than separate one-year studies only.

During the last decade a variant form of scrapie, Nor98, has been diagnosed in sheep in most countries in Western Europe including Iceland (Materials and methods). The Nor98 variant is different from classical scrapie in several ways. Of special concern is that sheep with genotype ARR/ARR considered resistant to classical scrapie are fully susceptible to the Nor98 variant [28]. Furthermore the occurrence of Nor98 scrapie might, in contrast to classical scrapie, be spontaneous and not, or at least less infectious in nature [29,30]. It thus seems necessary in future scrapie research to define as far as is possible the type of scrapie under study.

It was concluded that: 1) Fe tended to be in lower amounts in sheep forage in scrapie-free than in endemic areas; 2) Mn was in higher amounts in sheep forage in scrapie-free than in endemic areas; 3) the reciprocality between Fe and Mn results in lower Fe/Mn ratios in scrapie-free areas than in endemic areas and the observed ratios may possibly be taken as an index of scrapie status; 4) any relation between Cu and Zn levels in the forage and the occurrence of scrapie is unlikely; 5) the levels of Cu and especially Zn in sheep forage are seemingly lower than optimal in Iceland; 6) further study on the possible role of Fe and Mn in relation to the occurrence of scrapie in Iceland is clearly warranted.

## Competing interests

The authors declare that they have no competing interests.

## Authors' contributions

All authors contributed equally to the research. All authors read and approved the manuscript.
